# Grape ASR-Silencing Sways Nuclear Proteome, Histone Marks and Interplay of Intrinsically Disordered Proteins

**DOI:** 10.3390/ijms23031537

**Published:** 2022-01-28

**Authors:** Hristo Atanassov, Jonathan Parrilla, Caroline Artault, Jérémy Verbeke, Thomas Schneider, Jonas Grossmann, Bernd Roschitzki, Rossitza Atanassova

**Affiliations:** 1 UMR CNRS 7267 Écologie & Biologie des Interactions, Équipe, Sucres & Echanges Végétaux-Environnement, Université de Poitiers, 3 Rue Jacques Fort, 86073 Poitiers, France; hristo.atanassov@univ-poitiers.fr (H.A.); jonathan_parrilla@outlook.fr (J.P.); caroline.artault@univ-poitiers.fr (C.A.); jeremy.verbeke@uca.fr (J.V.); 2 CHU de Poitiers, 2 Rue de la Milétrie, 86021 Poitiers, France; 3 Institute of Plant Biology, University of Zurich, Zollikerstrasse 107, 8008 Zurich, Switzerland; thomas.schneider@biognosys.com; 4 Functional Genomics Center Zurich, University of Zurich and ETH Zurich, Winterthurerstrasse 190, 8057 Zurich, Switzerland; jg@fgcz.ethz.ch (J.G.); bernd.roschitzki@fgcz.uzh.ch (B.R.)

**Keywords:** ASR, grape embryogenic cells, histone PTMs, IDPs, iTRAQ, LEA D-29, nuclear proteome, *VvMSA*-RNAi silencing

## Abstract

In order to unravel the functions of ASR (Abscisic acid, Stress, Ripening-induced) proteins in the nucleus, we created a new model of genetically transformed grape embryogenic cells by RNAi-knockdown of grape *ASR* (*VvMSA*). Nuclear proteomes of wild-type and *VvMSA*-RNAi grape cell lines were analyzed by quantitative isobaric tagging (iTRAQ 8-plex). The most significantly up- or down-regulated nuclear proteins were involved in epigenetic regulation, DNA replication/repair, transcription, mRNA splicing/stability/editing, rRNA processing/biogenesis, metabolism, cell division/differentiation and stress responses. The spectacular up-regulation in VvMSA-silenced cells was that of the stress response protein VvLEA D-29 (Late Embryogenesis Abundant). Both *VvMSA* and *VvLEA* D-29 genes displayed strong and contrasted responsiveness to auxin depletion, repression of *VvMSA* and induction of *VvLEA D-29*. In silico analysis of VvMSA and VvLEA D-29 proteins highlighted their intrinsically disordered nature and possible compensatory relationship. Semi-quantitative evaluation by medium-throughput immunoblotting of eighteen post-translational modifications of histones H3 and H4 in *VvMSA*-knockdown cells showed significant enrichment/depletion of the histone marks H3K4me1, H3K4me3, H3K9me1, H3K9me2, H3K36me2, H3K36me3 and H4K16ac. We demonstrate that grape ASR repression differentially affects members of complex nucleoprotein structures and may not only act as molecular chaperone/transcription factor, but also participates in plant responses to developmental and environmental cues through epigenetic mechanisms.

## 1. Introduction

Plant ASRs (Abscissic acid, Stress, Ripening proteins) have been discovered in tomato, as induced by water deficit in leaves and by ripening in fruit [1]. Identification of a plethora of ASRs in a multitude of higher plant species, gymnosperms and angiosperms (monocots and dicots), has confirmed their involvement in different stages of development (seed germination, flowering interval between female and male organs maturation, pollen desiccation, leaf senescence, fruit ripening), and in response to environmental cues (water, cold, salt and osmotic stresses, heavy metal and pesticide toxicity, and fungal diseases [2,3,4,5,6,7,8,9,10].

At a cellular level, ASR proteins have been localized both in cytoplasm and in the nucleus, which is due to the presence or the absence of a functional nuclear localization signal [11,12,13,14,15]. This dual subcellular location appears as a prerequisite for their functional duality. In fact, they supposedly act as molecular chaperones directly protecting biological macromolecules under stress, and noncanonical transcription factors in complexes for gene expression regulation [11,13]. It has been shown that ASRs, as highly hydrophilic proteins, display the ability to maintain certain enzymatic activities, such as those of lactate dehydrogenase and malate dehydrogenase, after several freeze/thaw cycles [16] and heat treatment [17]. In this regard, the heterologous overexpression of tomato ASR1 in potato has suggested its possible involvement in the regulation of glucose metabolism and carbon reallocation [18]. Furthermore, the antisense repression of *SlASR1* in transgenic tobacco plants has clearly demonstrated a decrease in CO_2_ assimilation, increased diurnal accumulation of glucose in leaves concomitant with a significant reduction of sucrose in phloem sap, and diminished expression of hexose transporter HT1 and sucrose transporter SUT2 [19].

The grape ASR, VvMSA, has been identified as directly involved in gene expression regulation of the glucose transporter VvHT1, at the convergence of sugar and abscisic acid signaling pathways [11]. A model of the fine-tuning of VvMSA transcription regulation at promoter level by glucose and ABA has been built through the interplay of Hexokinase 1 (HXK1) and Sucrose-nonfermenting Related Kinase 1 (SnRK1) [20]. The role of ASRs at the interface of sugar metabolism and hormone signal transduction pathways, as well as their impact on the control of plant growth, development and response to environmental constraints, has been reported [19,21].

ASRs have been classified as the seventh group of the large family of LEAPs (Late Embriogenesis Abundant Proteins), themselves belonging to the Dehydrins superfamily [22]. Four ASRs—tomato SlASR1, plantain MpASR, barley HvASR and wheat TtASR—have been considered Intrinsically Disordered Proteins (IDPs) [23,24,25]. Previously, it has been reported that IDPs lack a well-defined structure in their native state and under physiological conditions in the absence of a partner, in terms of protein/protein or protein/nucleic acid interactions [26,27,28,29,30]. Tomato SlASR1 has been demonstrated to adopt ordered conformation in the presence of Zn^2+^, thereby favoring its fixation to DNA [12]. Moreover, SlASR1 has been involved in the formation of homodimers and homotrimers capable of interacting with DNA [14,31]. The grape ASR belongs to the histone nucleosomal fraction of nuclear proteins and is entangled in a protein heterodimer with VvDREB, an APETALA2 transcription factor (TF), thus acting as a cofactor of architectural type involved in the recruitment of another canonical TF within a complex for transcriptional regulation [32]. Very recently, this finding has been corroborated by the demonstration that *Brachipodium distachyon* BdASR1 is able to interact with another member of the same APETALA2/Ethylene Responsive Factor (AP2/ERF) superfamily, the BdERF 96, in the plant response to drought and oxidative stresses [33]. In addition, several genes have been revealed as direct ASR targets by chromatin immunoprecipitation-based sequencing (ChIP-seq) using antibodies to tomato SlASR1 [34] and banana MaASR overexpressed in *Arabidopsis* [35]. In rice, an ASR (OsASR5) has been found to be involved in the regulation of a microRNA gene expression, i.e., *osa-MIR167a* [36].

Despite experimental evidence provided by different approaches for studying the interactions of ASR proteins with DNA (gel shift assay, yeast one-hybrid screening, in planta co-expression experiments, ChIP-seq) in tomato, grapevine, rice [11,15,37,38] and with other nuclear proteins [32,33], the precise biological roles of ASR proteins in the nucleus remain elusive and necessitate further elucidation.

To shed more light on this issue, we succeeded in the genetic transformation of embryogenic grape cells and the RNAi-silencing of *VvMSA*, and used this model to compare the nuclear proteome of wild-type cells expressing the grape ASR, and *VvMSA*-RNAi-silenced cells. To that aim, we performed eight-plex iTRAQ (isobaric Tag for Relative and Absolute Quantification) of proteins differentially extracted from isolated nuclei. The *VvMSA*-silencing significantly affected the expression level of 146 nuclear proteins involved in epigenetic, transcriptional, post-transcriptional and translational control of plant responses to developmental and environmental cues. The remarkable induction of one LEA protein in the absence of VvMSA, and their antagonistic relationship under auxin depletion in grape embryogenic cells, confirmed the interplay between these IDPs. To further explore the impact of VvMSA silencing on epigenetic landscape, we developed custom medium-throughput immunoblotting assay of multiplex type, applied at the level of total chromatin, and revealed significant quantitative changes in several H3 and H4 histone post-translational modifications in the absence of grape ASR.

## 2. Results

### 2.1. Nuclear Proteome of Grape Wild-Type and VvMSA-RNAi-Silenced Cells

**Figure 1:**
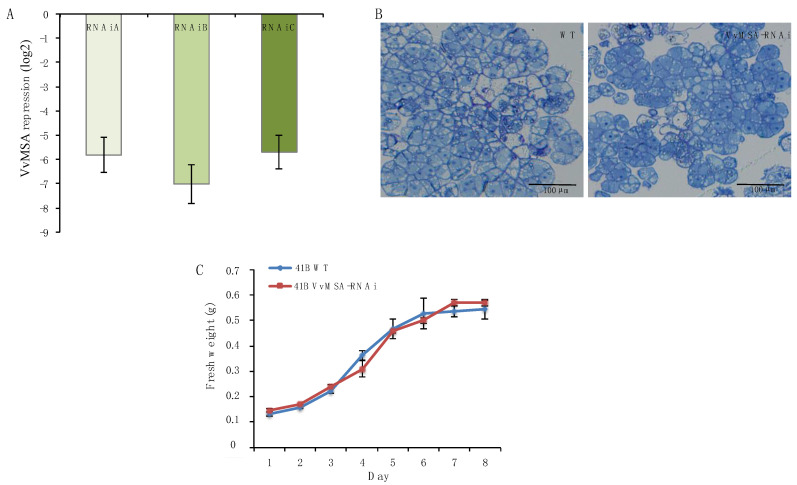
Phenotypic characterization of grape embryogenic 41B cells: wild-type and *VvMSA*-RNAi. (**A**) Real-time qPCR analysis of the grape ASR gene repression in three independent *VvMSA*-RNAi transgenic lines. The expression of each *VvMSA*-RNAi line was reported to that of the control wild-type cells (previously normalized to the reference *VvACT* gene). The repression of *VvMSA* was calculated by the 2^−∆∆Ct^ method, and presented as log2 fold change. The results correspond to the mean value and the standard error of three biological replicates for each cell line. (**B**) Cell morphology observation by light microscopy after toluidine blue staining (Olympus DP72): wild-type (left) and *VvMSA*-RNAi (right)). (**C**) Growth curves of wild-type cells (blue line) and *VvMSA*-RNAi transgenic cells (red line).

Three fully independent transformation experiments were carried out on grape embryogenic cells 41B, at three different time periods and by three manipulators, using exactly the same protocol for genetic transformation. *VvMSA* silencing in the three transgenic cell lines was tested by real-time qPCR (Figure 1A), which confirmed the successful RNA-interference knockdown of this grape ASR. It is worth noting that VvMSA repression in 41B embryogenic cells affected neither cell morphology (Figure 1B), proliferation capacity (Figure 1C), nor their differentiation ability for somatic embryogenesis, which was confirmed by the regeneration of *VvMSA*-RNAi silenced plantlets (Figure S1).

The originality of this cellular model consists in the homogeneity and the relative synchronization of the grapevine embryogenic cell population, thus circumventing one of the most critical problems in proteomic and epigenetic investigations due to plant cell heterogeneity within tissues. Consequently, transgenic versus wild-type embryogenic cells were chosen as a new model for studying ASR impact on nuclear proteome and histone epigenetic marks.

As far as we know, our study reports nuclear proteome analysis in grape for the first time. One of the features of iTRAQ is that this technology allows quantitative comparison of proteins, which must be present in both studied conditions. This explains why in our experiments VvMSA could not be identified by iTRAQ in the *VvMSA*-silenced cells, even though it is expressed in the wild-type cells.

Among all identified 484 nuclear proteins, 447 were successfully annotated in grapevine and only 37 remained with unknown function. A total of 146 proteins out of 484 displaying a 1.2-fold change and Q-value > 0.05 were selected as differentially expressed proteins (DEPs) between wild-type and *VvMSA*-RNAi silenced cells (Table 1). These 146 proteins with significant differential expression encompassed 137 up-regulated and 9 down-regulated in *VvMSA*-RNAi versus wild-type.

We first annotated the DEPs using BLAST to search for significant sequence homology and Uniprot to unravel conserved functional domains. As shown in Table 1, this approach allowed us to classify the proteins in eight functional groups: (1) cell division and differentiation (*n* = 11); (2) DNA replication and repair (*n* = 5); (3) epigenetic regulation (*n* = 29); (4) metabolism (*n* = 13); (5) mRNA splicing, stability and editing (*n* = 42); (6) rRNA processing and biogenesis (*n* = 13); (7) stress response (*n* = 13); (8) transcriptional regulation (*n* = 20).

The sequences of the DEPs were also submitted to functional enrichment analysis for protein–protein interactions using the network’s STRING database. This analysis suggested that 142 out of the 146 grape proteins could interact with other protein partners.

**Figure 2:**
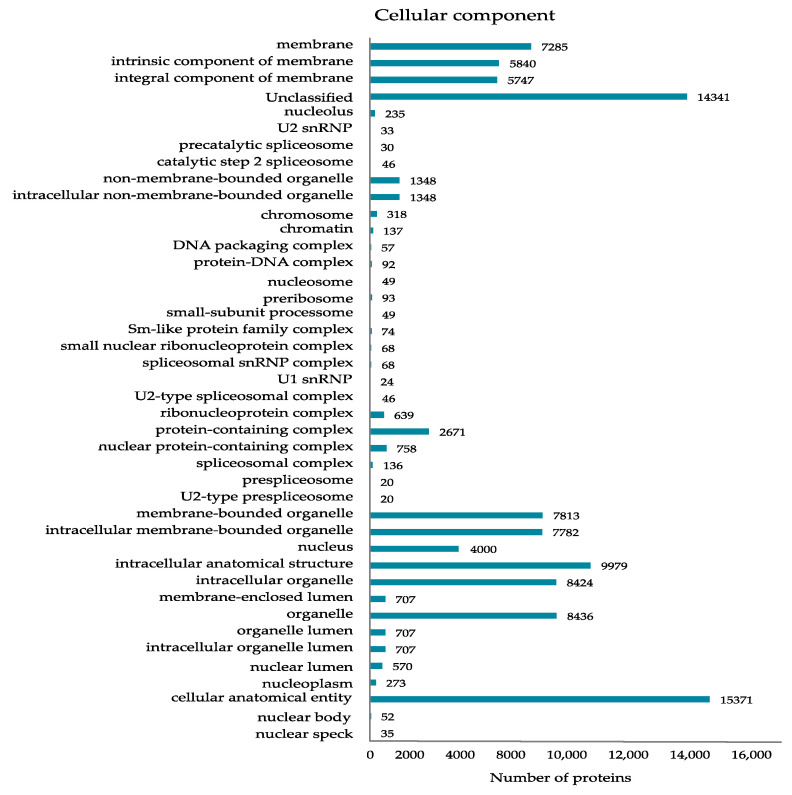
GO classification of the DEPs by localization as cellular components.

**Figure 3:**
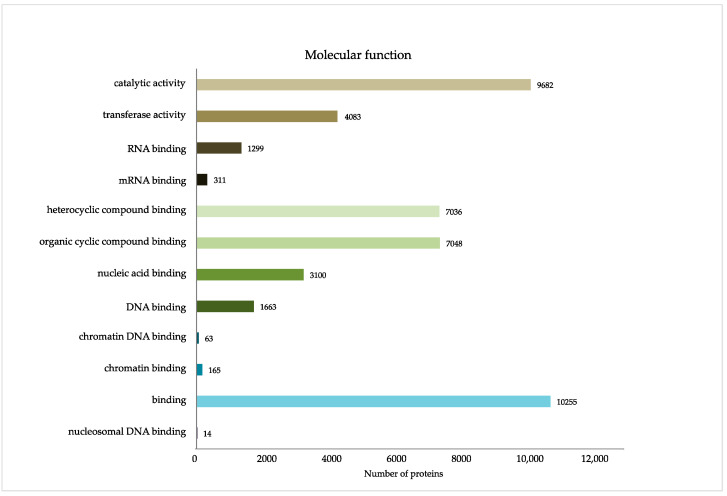
GO classification of the DEPs by molecular functions.

Gene Ontology (GO) enrichment analysis of the DEPs provided more details for their localization as cellular components (Figure 2), molecular functions (Figure 3), and involvement in biological processes (Figure S2). The latter classification turned out to be particularly exhaustive, suggesting involvement in more than hundred biological processes (Figure S2). The classification by cellular components confirmed the annotated DEPS as nuclear proteins, their localization in distinct nuclear subdomains, nucleoprotein- and protein–protein complexes (Figure 2). Interestingly, the most concise GO classification was generated when using the criterion of molecular function: only twelve molecular functions were suggested so far, mainly of DNA, RNA and cyclic compound binding, as well as of catalytic and transferase activity (Figure 3).

### 2.2. Late Embryogenesis Abundant Protein VvLEA-D29

**Figure 4:**
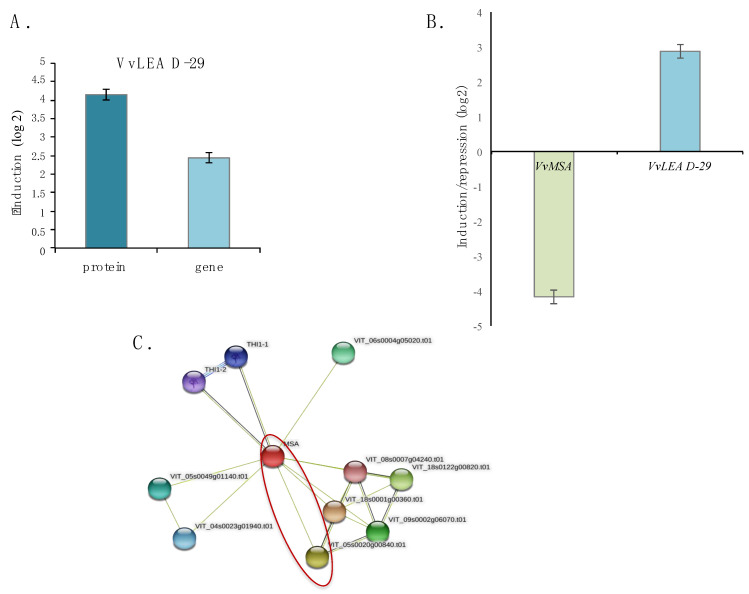
Relationship between VvMSA and VvLEA D-29 genes and proteins. (**A**) Induction of LEA D-29 protein in *VvMSA*-RNAi silenced cells (quantified by iTRAQ) and *LEA D-29* gene expression in *VvMSA*-RNAi cells (measured by RT-qPCR). The expression of *LEA D-29* gene in *VvMSA*-RNAi cells was reported to that of the control wild-type cells (previously normalized to the reference *VvACT* gene) and the induction of *LEA D-29* (presented on the figure) was then calculated by the 2^-∆∆Ct^ method, as log2 fold change (mean ± SE). (**B**) Down-regulation of *VvMSA* and up-regulation of *VvLEA D-29* genes by auxin depletion at the 4th day after somatic embryogenesis induction of wild-type 41B cells (three biological repetitions). The expression of each gene under auxin depletion (previously normalized to the reference *VvACT* gene) was reported to that of 41B cells cultured into auxin-supplemented medium by using the 2^−ΔΔCt^ method (mean ± SE). (**C**) Relationship between grape ASR (MSA; VIT_18s0072g00380.t01) and Late Embryogenesis Abundant protein LEA D-29 (VIT_05s0020g00840.t01) established by using the protein–protein interaction and functional enrichment network STRING (https://string-db.org/; accessed on 1 May 2021) in *Vitis vinifera*.

Our proteomic analysis revealed VvLEA D-29 as the most affected protein by the *VvMSA* silencing, which displayed a log2 fold change of 4.17 responding to nearly 18-fold up-regulation in *VvMSA*-RNAi-A cells compared to that of the wild-type cells (Figure 4A). This was further confirmed by the significant overexpression of *VvLEA D-29* gene in the same transgenic *VvMSA*-RNAi-A cells, as demonstrated by real-time qPCR (Figure 4A). Despite the strong increase at both protein and gene levels, the protein displayed a higher induction when compared to that of the gene, which highlights the importance of post-transcriptional regulation in the accumulation of LEA protein in grape *VvMSA*-RNAi cells. Because of the embryogenic nature of the grape 41B cells, *VvMSA* and *VvLEA D-29* expression was further analyzed under conditions of initial triggering of somatic embryogenesis by auxin depletion of the culture medium. Both genes demonstrated strong and contrasted responsiveness to auxin depletion, nearly 18-fold down-regulation of *VvMSA* and more than 7-fold up-regulation of *VvLEA D-29* (Figure 4B). Eventually, in silico STRING analysis for protein–protein interaction and functional enrichment provided additional argument in favor of the plausible relationship between VvMSA and VvLEA D-29 (Figure 4C).

**Figure 5:**
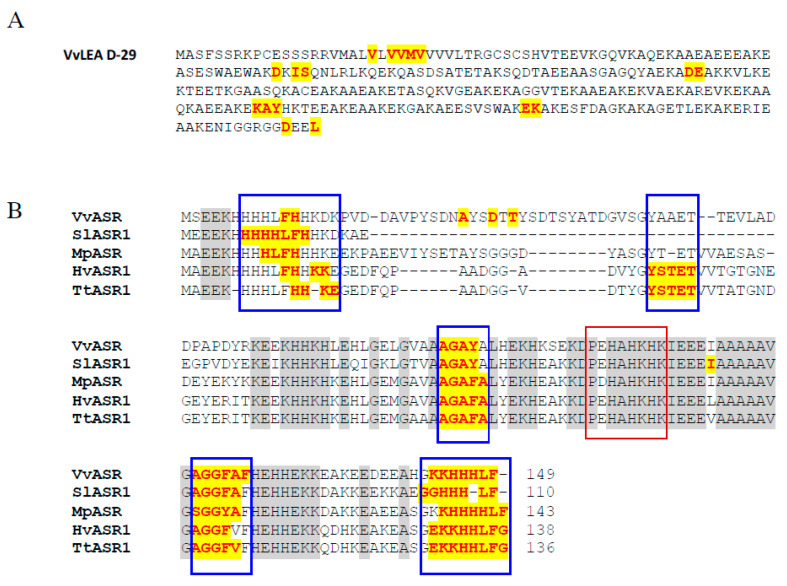
Prediction of the intrinsically disordered regions by MoRFpred. (**A**) Identification of Molecular Recognition Elements (MOREs) in the sequence of VvLEA D-29. (**B**) Multiple sequence alignment of five ASRs with their predicted intrinsically disordered regions: VvMSA (*Vitis vinifera*); SlASR (*Solanum lycopersicum*); MpASR (*Musa* ABB Group); HvASR1 (*Hordeum vulgare*); TtASR1 (*Triticum turgidum* subsp. *Durum*). The amino acids in bold, red, and highlighted in yellow denote the short disorder-to-order transitioning binding regions (blue frame). The amino acids in normal, black and highlighted in gray are identical in all of the five ASR sequences. The Zn^2+^-binding region PEHAHKHK (red frame) is identical in grape, tomato, plantain, barley and wheat.

As most members of the LEA superfamily are either partially or entirely IDPs [39,40], we checked the presence of short clusters enriched in hydrophobic amino acids corresponding to Molecular Recognition Elements (MOREs) in the sequence of VvLEA D-29 by MoRFpred prediction. Grape LEA D-29 encompasses several disordered regions in its amino acid sequence, and consequently, it may also be considered as partially disordered (Figure 5A). Furthermore, we looked for structural disorders in the primary sequence of grape ASR. The in silico analysis of disordered regions of grape ASR and their comparison with four characterized ASR proteins from tomato, banana, barley and wheat [23,24,25] brought evidence for the presence of five almost identical regions of disorder within the VvMSA sequence (Figure 5B). The zinc-induced gain of structure also results in a conformational transition and, consequentially, in decreased susceptibility to trypsin digestion, as already reported for tomato ASR1 [23,41]. The Zn^2+^-biding region (PEHAHKHK), previously identified in tomato ASR, is also conserved in the other ASR proteins that are characterized as IDPs (Figure 5B).

### 2.3. Impact of VvMSA Repression on H3 and H4 Histone Post-Translational Modifications (HPTMs)

**Figure 6:**
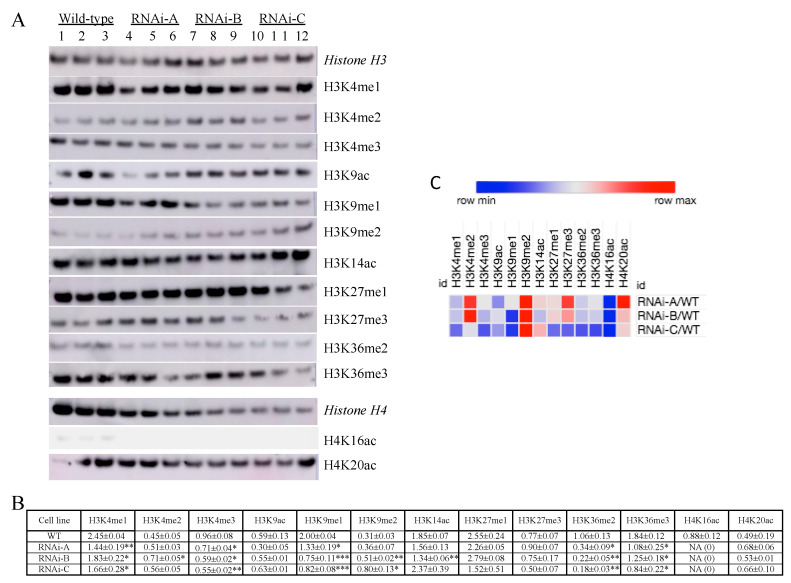
Immunoblot analysis of histone H3 and H4 PTMs at the level of the total chromatin of wild-type and three independent *VvMSA*-RNAi transgenic lines (biological replicates) of grape embryogenic cells 41B. (**A**) Histone PTMs’ immunodetection of the four cell lines, each tested in three technical replicates. The loading controls stained with Ponceau S are presented in Figure S5. (**B**) The quantified HPTM data were first normalized to those of their respective native histone (H3 or H4). The values of the three technical replicates for each of the four tested cell lines were presented as mean ± SEM. The asterisks denote the level of significant difference between the wild-type and each of the RNAi cell lines evaluated by unpaired *t* test: * *p* < 0.05; ** *p* < 0.01; *** *p* < 0.001. In the column of H4K16ac statistics was not applicable (NA) because of undetectable signal in the three tested RNAi cell lines. (**C**) Heat map of the mean *VvMSA*-RNAi/WT ratios of the three biological replicates (Morpheus, https://software.broadinstitute.org/morpheus; accessed on 1 May 2021). The heat map was built using the mean *VvMSA*-RNAi/WT values presented in Table S3.

We have previously characterized the grape ASR as a transcription factor of the architectural type belonging to the chromatin fraction of nuclear proteins [32]. In parallel, twenty-nine proteins involved with epigenetic regulation displayed quantitative differences (Table 1), which implies shifts of post-translational modifications of the histones H3 and H4. The latter raises the pertinent question of whether *VvMSA* silencing affects histone marks. To that aim, we compared HPTM changes between the three generated independent *VvMSA*-RNAi transgenic cell lines (biological replicates) and the original wild-type cell line, each of them tested in three technical replicates. We developed a custom medium-throughput immunoblot assay of multiplex type coupled with ImageQuant TL analysis to simultaneously test immunodetection histone H3 and H4 PTMs versus a panel of twenty antibodies, using ECL. The used monoclonal primary antibodies were directed against eighteen HPTMs of lysine residues: twelve of histone H3 (nine methylations and three acetylations) and six of histone H4 (five acetylations and one methylation) (Figure 6A; Table S1). All data of quantified HPTMs were normalized to those of their respective histone by using monoclonal antibodies raised to synthetic peptide H3 and H4 whole sequences devoid of any HPTM.

In regard of histone H3 we detected statistically significant differences between the *VvMSA*-RNAi cells and the wild-type cells in six out of the eleven detected HPTMs: H3K4me1, H3K4me3, H3K9me1, H3K9me2, H3K36me2 and H3K36me3. No statistical difference was observed for H3K4me2, H3K9ac, H3K14ac, H3K27me1 and H3K27me3. One histone H3 PTM (H3K27ac) was not detected (Figure 6B). Concerning the histone H4, only two out of the six tested HPTMs were detected (i.e., H4K16ac and H4K20ac), while three other HPTMs were undetectable (H4K5ac, H4K12ac, H4K20me1) and the used antibody to H4K8ac produced multiple artefactual bands. Most importantly, H4K16ac was detected only in the wild-type 41B cells, which underlies its depletion in the three independently transformed *VvMSA*-RNAi cell lines (Figure 6B). The other marked changes in the *VvMSA*-RNAi cells consisted of a nearly two-fold increase in H3K9me2, two-fold decrease in H3K9me1 and five-fold decrease in H3K36me2 (Figure S5). The latter results were visualized on a heat map (Figure 6C, Table S3).

## 3. Discussion

ASR proteins, at the example of VvMSA, are expressed in the transitions between different stages of plant development and in plant responses to environmental cues, mediated by complex interplay of endogenous (hormonal and metabolic) and exogenous signals [21]. Each of these respective developmental transitions and adaptive responses is subject to strong metabolic changes, transduced by specific epigenetic modifications and consequent differential expression of distinct sets of genes (recently reviewed by Leung and Gaudin, 2020) [42]. In plants, the genes encoding transcription factors are favorite targets of epigenetic regulations, and are considered to represent nearly 15% of all protein coding genes. In addition, their combinatory effects on gene expression are further fine-tuned by the mechanisms of post-transcriptional control. Studies on plant processome/ribosome proteins provide evidence for their essential role in regulation of plant development [43,44,45] and response to environmental stresses [46,47,48]. Another level of complexity of plant ribosomal proteins is dealing with the existence of several paralogues displaying divergent functions due to their post-translational modifications (acetylation and phosphorylation). Such additional functionalization appears dependent on genetic or epigenetic factors and provides sub-specialization of the different ribosomal proteins allowing adaptation of the plant response to environmental factors [48]. In this general context, the silencing of the only one ASR found in grape impacts nuclear proteome by up- or down-regulation of 146 proteins clustered in the functional groups of metabolism, epigenetic regulation, DNA replication and repair, transcriptional regulation mRNA-splicing, stability and editing, rRNA processing and biogenesis, cell division and differentiation, and stress response (Table 1).

In our study the generated *VvMSA*-RNAi silenced cells and their control, the WT cell line, were chosen as an appropriate model because of the lack of apparent differences in terms of cell morphology, proliferation rate and differentiation potential. It is worth noting that the glucose absorption by *VvMSA*-RNAi silenced cells decreased nearly two-fold in comparison to the WT cells (our unpublished results). The latter corroborates the idea that the repression of grape ASR affects plant metabolism, and this in the absence of apparent phenotypic alterations (Figure 1B,C). In this regard, we have already reported that 41B embryogenic cells cope with the low level of intracellular glucose and the low glycolysis efficiency still capable of sustaining their organized cell proliferation [49]. Furthermore, genetically modified plantlets, regenerated from transgenic somatic embryos, did not display apparent phenotypic differences when compared to those of the wild-type plantlets cultured under the same heterotrophic conditions (Figure S1). Although these transgenic grape plantlets may be “indefinitely” micro-propagated under heterotrophic conditions, they were not able to acclimate to autotrophic conditions. The failure of acclimation of grape ASR-silenced plantlets from heterotrophic to autotrophic conditions represented the most marked phenotypic difference when compared to in vitro regenerated WT plantlets. Such an issue may be explained by the roles of ASRs in transcriptional regulation of some sugar transporter genes, glucose metabolism and glucose signaling [11,16,18]. In the latter regard, Dominguez and co-authors have already demonstrated the crucial impact of antisense reduced expression of tomato ASR1 on the decrease in CO_2_ assimilation and sucrose loading in phloem, concomitant with the increase of glucose accumulation in leaf mesophyll cells, at the crosstalk between sugar, ABA and gibberellin signaling pathways. They have also provided evidence for partial degradation of large and small Rubisco subunits, as well as for induced production of oxygen reactive species as marks of accelerated developmental senescence due to glucose accumulation in leaves and related glucose signal transduction via its cytosolic sensor the HXK1 [19].

One of our conspicuous findings concerns the fact that after VvMSA repression the majority of DEPs turn up-regulated, which emphasizes the critical functions of the ASRs in plant developmental transitions and stress responses. Interestingly, the most up-regulated protein in our study, VvLEA D-29, belongs to the superfamily of the late embryogenesis abundant proteins (LEAPs). These highly hydrophilic proteins are considered to play a crucial role in plant adaptive response, at the onset of abiotic stresses induced by low temperatures (cold and freezing), dehydration, and salinity [22,50]. The LEAPs have initially been discovered to accumulate in the last phases of embryo development, as protection against protein aggregation under seed desiccation. In the latter context, it should be pointed out that our present results were obtained on grape embryogenic 41B cells, mainly used for genetic transformation through differentiation of transgenic somatic embryos.

The grape LEA D-29 (VvLEA D-29) protein displaying LEA4 domain has been identified in grapevine as the unique member of subclass 4 of the large LEA family [51]. The nucleo-cytosolic localization of VvLEA D-29 has been demonstrated, and its encoding gene has been characterized in two different grape cultivars as up-regulated by salinity and PEG-induced osmotic stress [51]. The strongly induced expression of VvLEA D-29 under grape ASR repression, the abiotic stresses responsiveness and the nuclear localization of these two proteins, argue in favor of a possible compensatory effect of VvLEA D-29 to VvMSA silencing. It is worth mentioning that the specific silencing of rice *ASR5* by microRNA approach has been compensated by concomitant induction of rice *ASR1* [15]. In addition, it has been demonstrated that the functional complementarity of both of these OsASR proteins erases the dwarf phenotype due to the simultaneous RNAi silencing of *OsASR1* and *OsASR5*, and OsASR1 expression perfectly restores the normal phenotype under OsASR5 depletion [8,15]. Even though grape ASR and grape LEA 29-D belong to two different groups of LEAPs, our results suggest a plausible compensatory effect between them under *VvMSA*-silencing and auxin depletion.

Aberrant electrophoretic migration as another characteristic trait of protein disorder has already been demonstrated for VvMSA (theoretical MW of 16.5 kDa), whose MW after denaturing electrophoresis is estimated at 23.4 and 24 kDa for the deleted and the complete forms, respectively [11,32]. The extremely high sequence homology of disorder responsible regions, shared by the grape ASR and the four above-mentioned ASR proteins, as well as its aberrant electrophoretic mobility, allowed us to predict VvMSA as a potential IDP. The IDPs have been described as highly dynamic and conformational heterogeneous structures showing a propensity to undergo induced partial folding upon binding to a partner or under constraint non-physiological conditions [52,53,54]. The disordered nature of the four above-mentioned ASRs highlights structural similarities between ASR and LEA, and corroborates the classification of ASR proteins as a subfamily of the LEAPs superfamily [22,49,55,56]. A hypothesis, whose veracity has yet to be tested, predicts many LEAPs as positively associated with stress memory in Arabidopsis [57].

As the functional base of stress memory relies on different epigenetic modifications, we further explored HPTMs at total chromatin level. At the current state of knowledge, the challenge consists of deciphering the causal relationship between changes in HPTMs and those at transcription level. The transcriptional machinery is strongly dependent on local high-ordered structures of chromatin, which determines its accessibility and represents a biologically active platform for complex nucleic acids and proteins interactions. In other terms, the chromatin structural context tightly impacts the effectiveness of transcriptional regulation, and thereby its remodeling controls gene expression. The genome-wide analysis of histone marks and their plotting to transcription activity have revealed H3K4me3 and H3K36me3 post-translational modifications as positive marks of actively transcribed genes, and H3K27me3 as a negative mark of transcriptionally inactive genes [58,59,60,61]. Consequentially, these HPTMs have been related to two different chromatin states (CS): CS1 enriched with H3K4me3 and H3K36me3 for genes of high transcript level, and CS2 enriched of H3K27me3, often associated with genes of low transcript level, both CS concerning the euchromatin [60].

In our histone PTMs analysis of total chromatin, the quantitative modifications of H3K4me3 and H3K36me3 were reduced by more than 30% in the *VvMSA*-RNAi cell lines. This partial depletion of both histone marks argues in favor of a relative reduction in actively transcribed genes in *VvMSA*-silenced cells under normal growth. H3K4me3, as the most studied methylation mark in abiotic stress conditions, has been proposed as responsible for a memory effect during repeated stress exposure [62]. Furthermore, the dynamics of H3K4me2 and H3K4me3 enrichment have already been observed at the promoter region and the first exon of some immunity genes triggered by pathogen molecular patterns (*WRKY53*, *FRK1* and *NHL10*) after priming with mild abiotic stresses (i.e., heat, cold, salt) [63]. It seems therefore enticing to deduce that the depletion of grape ASR, which is strongly involved in plant stress responses, may affect the stability of multiprotein complexes responsible for chromatin remodeling.

H3K9me2, described as a feature of silent transposable elements and other repeats of repressive heterochromatin, has been associated with DNA methylation and appears characteristic for the chromatin state 3 [60]. H3K9me2 has been lost in tomato roots under drought conditions [64]. Inversely, in our HPTM analysis of total chromatin H3K9me2 displayed a nearly two-fold increase, with collective significance for all RNAi lines (A, B, C) and their technical replicates in comparison to the wild-type control (Figure 6B). This significant enrichment of H3K9me2 mark might suggest sustained silencing of transposons in the absence of stress-responsive VvMSA protein. In *Arabidopsis* seedlings subjected to heat stress, a *copia*-type retrotransposon named *ONSEN* has turned not only transcriptionally active, but also has generated novel stress-responsive regulatory genes [65]. Activation of another transposon named *Athila* leads to the production of small RNAs that in turn regulate expression of a key gene involved in stress tolerance [66]. Taken together with our results on H3K9me2, these examples of reactivated transposable elements highlight a novel putative role of VvMSA in the reduced grape cell responsiveness to abiotic stresses.

Eventually, H4K16ac was detected only in the wild-type cell line expressing VvMSA, while this histone mark was not observed in the three independently transformed *VvMSA*-RNAi cell lines. Studies in yeast, Arabidopsis and rice have already revealed differential functions for the acetylation at this specific position, lysine 16 of histone H4 [67,68]. Hyperacetylation of H4K16 in budding yeast has appeared to be involved in the stability of heterochromatin boundaries and the high-order compaction of chromatin [69]. In Arabidopsis and rice, H4K16ac has been mostly enriched around the transcription start site, and its combined effect with H3K23ac has been suggested as critical for tissue-specific and developmental regulation of gene expression [70].

ASR proteins are expressed at the transition between different stages of plant development (such as seed germination, leaf senescence, fruit ripening), and each of these transitions is subject to strong epigenetic control [71]. Consequently, we may speculate the possible involvement of ASRs as molecular chaperones/transcription factors in these epigenetically regulated events. Another argument in favor of this hypothesis has been provided by the finding that rice ASR5 is not only able to recognize binding sites upstream of the microRNA gene (MIR167a), but also to drive its expression in vivo [36]. As microRNAs are key actors of gene expression that guide post-transcriptional control of plant development and responses to environmental stresses, and as microRNA genes have been identified as preferential targets of epigenetic regulation [72], it could be suggested that ASR proteins are involved in epigenetic regulation of gene expression.

## 4. Materials and Methods

### 4.1. Cell Culture and Transformation Conditions

The grapevine embryogenic cell line 41B was obtained from the most commonly used rootstock in the vineyards of Champagne (a hybrid between *Vitis vinifera* L. cv. Chasselas × *Vitis berlandieri* P.). The embryogenic cell suspension was subcultured every two weeks by transferring 0.3 mL of packed cell volume into 25 mL of a half-strength MS medium (Duchefa M0232) containing glycerol (4.6 G·L^−1^) and maltose (18 g·L^−1^) as carbon sources, as well as naphthoxyacetic acid (1 mg·L^−1^) and casein acid hydrolysate (Sigma A2427). They were cultured under constant agitation (110 rpm), in darkness and at 21 °C. In order to silence the grape ASR gene (*VvMSA*), 41B embryogenic cells were transformed with the *35S::VvMSA*-RNAi construct via *Agrobacterium tumefaciens* strain EHA 105, co-culture of the grape 41B cells with the bacteria, for 60 h on the above-mentioned solid medium, and selection of transformed cells on paromomycin (2 µg·mL^−1^) [73]. The somatic embryogenesis of 41B cells was induced by their subculture into the same fresh medium depleted of auxin and at 26 °C.

### 4.2. Real-Time qPCR Analysis

Reverse transcription was carried out on 1 µg of DNase-treated total RNA according to manufacturer protocol (Promega, Madison, WI, USA). Real-time qPCR was performed in 15 µL reaction mixture (5 µL of 10-fold diluted cDNA, and 10 µL of GoTaq^®^ PCR Master Mix 1X containing 0.375 µM of each primer), applying the program (2 min at 95 °C, followed by 40 cycles with 15 sec at 95 °C and 1 min at 60 °C), and using a Realplex^2^ Mastercycler (Eppendorf). The grape *Actin* gene was used as a reference. The primer sequences of the three genes are: *VvMSA* F: GCATGTGTGCTTGTTGTGTAA and R: TCACAAGGACACACAGAGAGA; *VvACT* F: GCATCCCTCAGCACCTTCCA and R: AACCCCACCTCAACACATCTCC; *VvLEA D-29* F: GCTTTGAACTGTCTGCCTCTT and R: CTCATTTGCGATAAGGATAAGG.

### 4.3. Isolation of Nuclei and Extraction of Nuclear Proteins

Isolation of nuclei and extraction of nuclear proteins were carried as already described [29] and detailed in Figure S3. The integrity and the enrichment of nuclei was controlled by epifluorescence microscopy after staining with Hoechst 33258 (Sigma-Aldrich, St. Louis, MO, USA). In order to yield maximum proteins and preserve their integrity, the extraction was designed to produce three consecutive fractions: NaCl-fraction of nucleosolic proteins with 10 mM Tris-HCl, pH 7.5 containing 150 mM NaCl, H_2_SO_4_-fraction of chromatin loosely bound proteins with 0.4 N H_2_SO_4_, and SDS-fraction of chromatin-tightly bound proteins with 10 mM Tris-HCl, pH 7.5 containing 1% SDS, all supplemented with protease inhibitors as described for the nuclei extraction. After precipitation the pellets of all three fractions were subjected to successive washes, two with 96% ethanol, two with 100% acetone, and after acetone evaporation proteins were preserved at −20 °C.

### 4.4. Experimental Design, Relative Quantification of Protein Abundance and Statistics

Four independent biological replicates from three nuclear protein fractions of wild-type and *VvMSA*-RNAi lines were sequentially extracted. These 24 protein extracts were individually digested and labeled using iTRAQ-8plex (Figure S4). The resulting peptides were further fractionated using SCX into 8 master fractions per extraction method. The rationale for the number of samples in an iTRAQ-8plex experiment was provided by the number of labels (*n* = 8) and the comparison of two groups. The quantitative protein ratios were normalized in Mascot by the median ratio. Ratios with *p* < 0.05 and fold changes > 2.0 were considered as significant. The identified proteins were submitted to *t*-test, normalized by mean values and eventually validated by Bonferroni test.

### 4.5. Protein Digestion, iTRAQ 8-Plex Labeling, and Peptide Fractionation

iTRAQ 8-plex experiments were performed to analyze the three nuclear protein fractions (“NaCl”, “H_2_SO_4_”, “SDS”) from four biological replicates obtained in two conditions of the embryogenic grape cell line 41B: wild-type (WT) and *VvMSA*-RNAi-A. Proteins reconstituted directly in 50 µL of 500 mM triethylammonium bicarbonate buffer (TEAB), pH 8.5), 50 µg per sample, were used for each iTRAQ channel. Tryptic digestion (10% *w*/*w*, sequencing-grade modified trypsin, Promega, Madison, WI, USA) and iTRAQ 8-plex labeling (SCIEX, Concord, ON, Canada) were performed according to the manufacturers’ instructions (16 h—trypsin digestion at 37 °C and 2.5 h—incubation of samples with respective iTRAQ labels). The iTRAQ labels for WT were 113, 116, 117 and 121; those for *VvMSA*-RNAi-114, 115, 118 and 119. After iTRAQ labeling, the samples were combined, desalted on 500 mg SepPak C_18_ columns (Millipore, Billerica, MA, USA), dried in a SpeedVac concentrator (ThermoFisher Scientific, Waltham, MA USA) and subjected to peptide fractionation by strong cation exchange chromatography (SCX). The samples were injected by using an autosampler (Agilent 1100 series, Agilent Technologies, Santa Clara, CA, USA) and directly loaded onto a 2.1 mm × 200 mm SCX-column (Poly-SULPHOETHYL A, 5 µm, 300-Å, PolyLC, Columbia, MD, USA). The peptides were eluted at a flow rate of 0.3 mL/min by using the following gradient: 0–10 min, 0% solvent B, 10–50 min, 0–35% solvent B; 50–65 min, 35–100% solvent B. Solvent A contained 10 mM KH_2_PO_4_ and 25% acetonitrile and solvent B—10 mM KH_2_PO_4_, 25% acetonitrile, and 0.5 M KCl; the pH of both buffers was adjusted to less than 3. In this way, the labeled peptides were separated into 54 fractions that were further pooled into 8 master fractions (according to the SCX spectrum) and purified using a C_18_ column (Sep-Pak cartridge, Waters Corporation, Milford, MA, USA).

### 4.6. Liquid Chromatography and Tandem Mass Spectrometry

Peptide samples of the pooled 8 master fractions from previous SCX chromatography (4 μL) were analyzed on an LTQ-Orbitrap Velos mass spectrometer (Thermo Fischer Scientific, Bremen, Germany) coupled to a nano-HPLC system (Eksigent Technologies, Dublin, CA, USA). The solvent compositions were 0.2% formic acid and 1% acetonitrile for channel A and 0.2% formic acid and 80% acetonitrile for channel B. Peptides were loaded onto an in-house made tip column (75 μm × 80 mm) packed with reverse-phase C_18_ material (AQ, 3 μm, 200 A, Bischoff GmbH, Leonberg, Germany) and eluted (flow rate, 250 nL/min; solvent B gradient: from 3 to 30% in 62 min, from 30 to 45% in 70 min, and from 45 to 97% in 75 min). Full-scan MS spectra (300–1700 *m*/*z*) were acquired at a resolution setting of 30,000 at 400 *m*/*z* after accumulation to a target value of 1 × 10^6^. For the eight most intense signals per cycle above a threshold of 1000, both collision-induced dissociation (CID) and higher-energy collisional dissociation spectra were acquired in a data-dependent manner. CID scans were recorded in the ion trap (settings: normalized collision energy, 35%; maximum injection time, 50 ms; automatic gain control, 1 × 10^4^ ions). For the higher-energy collisional dissociation scans, spectra were recorded at a resolution setting of 7500 at 400 *m*/*z* (normalized collision energy, 45%; maximum injection time, 125 ms; automatic gain control, 5 × 10^4^ ions). Charge state screening was enabled and singly charged states were rejected. Precursor masses previously selected for MS/MS were excluded from further selection for 60 s, and the exclusion window was set at 10 ppm. The maximum number of entries in the exclusion list was set at 500. The MS–MS runs of all eight pooled master SCX fractions of the “NaCl” samples and the first four pooled master SCX fractions of the “H_2_SO_4_” and the “SDS” samples were analyzed in duplicates, where precursors selected in the first run were excluded from fragmentation in the second run. The exclusion list was set on a time window of 4 min and a mass width of 10 ppm. Orbitrap spectra were acquired using internal lock mass calibration on *m*/*z* 429.088735 and 445.120025.

### 4.7. Peak List Generation and Database Search

Mascot Distiller 2.4.3.3 (Matrix Science, Boston, MA, USA) was used to generate Mascot generic format peak lists. Deisotoping and peak picking were not performed between 112.5 and 121.5 *m*/*z* (the range containing iTRAQ reporter ions), and the higher-energy collisional dissociation and collision-induced dissociation spectra were merged by summing the two scans from the same precursor [74,75]. For each nuclear protein fraction (NaCl-nuclesolic; H_2_SO_4_-chromatin loosely bound; SDS-chromatin tightly bound) all relevant Mascot generic format peak lists were concatenated and searched, using Mascot Server 2.3.02 (Matrix Science), against the grape protein database of Genoscope (http://www.genoscope.cns.fr/externe/GenomeBrowser/Vitis/; accessed on 1 July 2021) combined with 54,898 entries in Uniprot annotated to *Vitis vinifera*. That database was concatenated to its reversed decoyed FASTA database. The concatenated database contained a total of 162,488 proteins and 260 common MS contaminants. Methylthio (C), iTRAQ 8-plex labeling at the N terminus and lysine were set as fixed modifications, and variable modifications consisted of methionine oxidation, deamidation of asparagine and glutamine and iTRAQ 8-plex labeling of tyrosine. The isotope and impurity correction factors used for each iTRAQ label were those provided by the manufacturer. Precursor and fragment tolerances were set at 10 ppm and 0.8 Da, respectively. The enzyme specificity was set to trypsin with an allowance of up to one missed cleavage. Using Mascot internal export scripts, the transformed Mascot DAT files into XML files were parsed with in-house scripts so that peptide sequences, scores and intensities of the individual reporter ion channels were reported. Confidently identified and quantified peptides were selected with the following filters: rank 1 (best spectra assignment), ion score, >25. For the estimation of the false discovery rates at protein level, the formula in Käll et. al. (2008) was applied [76].

### 4.8. Immunoblotting Analysis

Acid-extracted nuclear proteins (H_2_SO_4_ fraction) of the four cell lines were separated by 1D SDS-PAGE under the following conditions: 2.5 µg protein load per 2.5 mm-wide lane of 1 mm-thick NuPAGE Novex bis-Tris 4–12% gels/format 26 well, run in triplicate in an XCell4 *SureLock*™ chamber filled with MES SDS running buffer, at 200 V constant, for 34 min, at RT. In an immediate next step, the separated proteins were submitted to Western transfer on 0.2 µm nitrocellulose membrane using a Power Blotter, at 5 A constant/25 V limit, for 5 min, at RT, and the quality of protein transfer was controlled by Ponceau S staining (Figure S5). The membrane was rinsed for 5 min in Tris-buffered saline—TBS (10 mM Tris, 150 mM NaCl, pH 8.0) supplemented with Tween-20 at 0.5% final concentration (0.5% TBST), blocked for 30 min with 2% TBST, rinsed in 0.05% TBST for 5 min, incubated for 1 h with the primary antibody diluted in 0.5% TBST, washed three times, 10 min each, in 0.05% TBST, incubated for 1 h with the secondary peroxidase-conjugated antibody diluted in 0.5% TBST, washed three times, 10 min each, in 0.05% TBST, and three more times, 5 min each in double distilled water, immediately overlaid with an *ex tempore* prepared mix of equal amounts of the Western blotting detection reagents A and B (ECL™ Prime, Amersham, Chicago, IL, USA; ref. RPN2232), at final volume 0.1 mL/cm^2^, incubated for 5 min, in darkness. All steps were carried out at room temperature. After exposure in a CCD camera (Amersham Imager AI600, GE Healtcare, Buckinghamshire, UK), the obtained ECL images were analyzed by using the software ImageQuant TL (GE Healthcare, Buckinghamshire, UK). The ECL signal intensity of each band was quantified after background subtraction and the raw quantitative data were directly exported for further statistical analysis by unpaired *t*-test using GraphPad Prism, Version 5. The data were visualized in a heatmap generated by the Morpheus software (https://software.broadinstitute.org/morpheus; accessed on 1 May 2021). References of PAGE and WB materials, user manuals, list of antibodies and their working dilutions are provided in Tables S1 and S2.

### 4.9. In Silico Protein Analysis

In silico protein analysis was performed by using tools for multiple sequence alignment (CLUSTAL O(1.2.4); https://www.ebi.ac.uk/Tools/msa/clustalo/; accessed on 1 May 2021), prediction of the intrinsically disordered regions (MoRFpred; http://biomine.cs.vcu.edu/servers/MoRFpred/; accessed on 1 May 2021) [77], and protein–protein interaction and functional enrichment (STRING: (Search Tool for the Retrieval of InteractiNG Genes/Proteins) https://string-db.org/; accessed on 1 May 2021).

## 5. Conclusions

In our pertinent model of grape embryogenic cells, the silencing of *VvMSA* by RNA-interference strongly impacts the nuclear proteome as revealed by iTRAQ-detected DEPs involved in epigenetic, transcriptional and post-transcriptional regulation, metabolism, cell proliferation and stress responses. The spectacular up-regulation of VvLEA D-29 protein in VvMSA-depleted cells, as well as their contrasted responsiveness to auxin depletion, implies a possible compensatory relationship of these IDPs. The demonstration that VvMSA repression differentially affects several members of distinct multiprotein and nucleoprotein structures, such as chromatin and its remodeling complexes, spliceosome, processome and ribosomes, argues in favor of a plausible role of grape ASR as a recruiting and/or stabilizing factor. Our data on the post-translational modifications of histones H3 and H4 in grape ASR-depleted cells corroborate the idea of VvMSA involvement in plant response to developmental and environmental cues through modulation of the epigenetic landscape.

## Figures and Tables

**Table 1:** Differentially expressed proteins in wild-type and *VvMSA*-RNAi grape embryogenic cells.

UniProtKB Accession	NCBI Accession	STRING Accession	Protein Function	*VvMSA*-RNAi vs. WT
**Cell division/Differentiation (n = 11)**				
F6GVS4 (F6GVS4 _VITVI)	CBI16879.3	VIT_14s0083g00450.t01	Protein FLX-like 2	1.78
D7TD96 (D7TD96_VITVI)	CBI28469.3	VIT_12s0057g01280.t01	G-strand specific single-stranded telomere-binding protein 2	1.24
F6HCE0 (F6HCE0_VITVI)	CBI25499.3	VIT_13s0067g00550.t01	Clathrin light chain 2-like	1.24
D7T0X4 (D7T0X4_VITVI)	CBI24194.3	VIT_19s0085g00200.t01	Stigma/style cell cycle inhibitor 1	1.28
F6H9W8 (F6H9W8_VITVI)	CBI22388.3	VIT_19s0085g00220.t01	Cell division cycle 5-like protein	1.22
D7SJS1 (D7SJS1_VITVI)	CBI15897.3	VIT_06s0004g07170.t01	Structural maintenance of chromosomes domain-containing protein	1.21
F6GZE1 (F6GZE1_VITVI)	CBI18813.3	VIT_00s0920g00020.t01	Sister chromatid cohesion protein PDS5 homolog B	1.28
D7U294 (D7U294_VITVI)	CBI36860.3	VIT_07s0005g00470.t01	Peter Pan-like protein	0.38
D7SJV2 (D7SJV2_VITVI)	CBI15928.3	VIT_06s0004g06870.t01	Proliferation-associated protein 2G4-like	0.33
D7T8K7 (D7T8K7_VITVI)	CBI26828.3	VIT_01s0011g05790.t01	Microtubule-associated protein 70-2	1.71
F6HJS7 (F6HJS7_VITVI)	CBI29537.3	VIT_00s0527g00030.t01	Pistil-specific extensin-like protein-like	1.78
**DNA replication/Repair (n = 5)**				
F6HAC8 (F6HAC8_VITVI)	CBI24290.3	VIT_06s0009g02520.t01	Replication factor C subunit 1-like	1.25
D7TIQ6 (D7TIQ6_VITVI)	CBI30132.3	VIT_08s0007g05120.t01	DNA gyrase subunit B	0.76
D7SH27 (D7SH27_VITVI)	CBI15707.3	VIT_17s0000g00910.t01	Histidine kinase-, DNA gyrase B-, and HSP90-like ATPase family	1.27
D7TDE2 (D7TDE2_VITVI)	CBI34931.3	VIT_01s0127g00840.t01	OB-fold nucleic acid binding domain containing protein	1.33
F6HSF7 (F6HSF7_VITVI)	CBI33677.3	VIT_14s0006g03280.t01	DNA double-strand break repair rad50 ATPase	1.48
**Epigenetic regulation (n = 29)**				
E0CRG0 (E0CRG0_VITVI)	CBI19114.3	VIT_18s0001g04770.t01	Apoptotic chromatin condensation inducer in the nucleus	0.71
A5BH86 (A5BH86_VITVI)	CBI30396.3	VIT_08s0007g02200.t01	High mobility group B protein 1 isoform X2	1.50
F6HUL8 (F6HUL8_VITVI)	CBI34365.3	VIT_02s0025g00090.t01	RNA-binding protein C25G10.01	1.53
D7T3I0 (D7T3I0_VITVI)	CBI25061.3	VIT_00s0179g00340.t01	Histone H2A.1	1.71
F6GV41 (F6GV41_VITVI)	CBI16181.3	VIT_06s0004g04230.t01	Histone H2B	1.74
D7U2L4 (D7U2L4_VITVI)	CBI36980.3	VIT_07s0005g01810.t01	Agenet domain-containing protein	1.87
D7TCM4 (D7TCM4_VIT	CBI27882.3	VIT_11s0016g01890.t01	Single myb histone	1.33
D7TED8 (D7TED8_VITVI)	CBI28861.3	VIT_12s0059g01310.t01	SUMO protein	1.43
D7TUZ2 (D7TUZ2_VITVI)	CBI34317.3	VIT_14s0030g00480.t01	RNA recognition motif family protein	1.26
D7SIC8 (D7SIC8_VITVI)	CBI15238.3	VIT_17s0000g06030.t01	Nucleosome/chromatin assembly factor group	1.28
F6I550 (F6I550_VITVI)	CBI39729.3	VIT_19s0015g00430.t01	DEK domain-containing chromatin associated protein	1.23
D7T5E7 (D7T5E7_VITVI)	CBI25730.3	VIT_00s0194g00020.t01	DNA-directed RNA polymerases IV and V subunit 4 isoform X1	1.41
A5BLU3 (A5BLU3_VITVI)	CBI15554.3	VIT_17s0000g02550.t01	Histone H1	1.39
D7TSR3 (D7TSR3_VITVI)	CBI33535.3	VIT_14s0006g01440.t01	Double-stranded RNA-binding protein 4-like	1.22
E0CQU8 (E0CQU8_VITVI)	CBI18902.3	VIT_18s0001g00660.t01	High mobility group B protein 9	1.27
D7U016 (D7U016_VITVI)	CBI35962.3	VIT_09s0002g02330.t01	Nucleosome assembly protein 1-like isoform 1	1.28
D7U469 (D7U469_VITVI)	CBI37645.3	VIT_04s0044g00110.t01	High mobility group B2 protein-like isoform 1	1.29
F6HDQ3 (F6HDQ3_VITVI)	CBI26253.3	Not available	Suppressor of gene silencing like protein	1.29
D7U7P2 (D7U7P2_VITVI)	CBI38821.3	VIT_15s0048g01290.t01	Histone deacetylase complex subunit SAP18	1.33
D7U4F5 (D7U4F5_VITVI)	CBI37552.3	VIT_04s0044g01140.t01	RNA-binding protein 8A	1.37
D7U5H5 (D7U5H5_VITVI)	CBI37994.3	VIT_03s0038g00620.t01	Zinc finger protein GIS2-like isoform 2	0.36
D7T8P4 (D7T8P4_VITVI)	CBI26865.3	VIT_01s0011g05360.t01	HMG-Y-related protein A	1.41
D7UB91 (D7UB91_VITVI)	CBI40015.3	VIT_15s0024g00620.t01	Chromo domain protein LHP1-like heterochromatin protein 1	1.43
F6GWG2 (F6GWG2_VITVI)	CBI17313.3	VIT_05s0029g00130.t01	High mobility group B protein 15-like	1.44
D7SN59 (D7SN59_VITVI)	CBI17088.3	VIT_06s0061g01240.t01	Histone deacetylase HDT1-like	1.48
F6HND0 (F6HND0_VITVI)	CBI31410.3	VIT_13s0019g04940.t01	Protein RNA-directed DNA methylation 3 isoform X1	1.58
D7UDB2 (D7UDB2_VITVI)	CBI40727.3	VIT_18s0122g01190.t01	High mobility group-like isoform 1	1.75
F6HTB7 (F6HTB7_VITVI)	CBI33920.3	VIT_02s0012g00870.t01	Nucleic acid binding protein	1.89
F6HIR3 (F6HIR3_VITVI)	CBI29042.3	VIT_10s0042g00830.t01	Lysine-specific demethylase 3B-like	1.92
**Metabolism (n = 13)**				
F6GSG7 (F6GSG7_VITVI)	CBI14856.3	VIT_17s0000g10430.t01	Glyceraldehyde-3-phosphate dehydrogenase, cytosolic	1.95
D7TGC8 (D7TGC8_VITVI)	CBI29552.3	VIT_00s0769g00010.t01	Peptidyl-prolyl cis-trans isomerase FKBP62	1.27
F6H4R0 (F6H4R0_VITVI)	CBI21690.3	VIT_19s0027g01660.t01	Peptidyl-prolyl cis-trans isomerase CYP59 isoform X1	1.68
D7U1R3 (D7U1R3_VITVI	CBI36679.3	VIT_05s0102g00560.t01	Peptidyl-prolyl cis-trans isomerase E	2.04
F6HTX9 (F6HTX9_VITVI)	CBI34281.3	VIT_14s0030g00950.t01	Superoxide dismutase [Cu-Zn]-like isoform 2	1.35
F6H0A2 (F6H0A2_VITVI)	CBI19970.3	VIT_18s0001g15570.t01	Acetyl-CoA carboxylase carboxyltransferase subunit beta	1.21
D7FBB2 (D7FBB2_VITVI)	CBI25114.3	VIT_16s0100g00580.t01	Nitrogen regulatory protein P-II homolog	1.23
D7TQA5 (D7TQA5_VITVI)	CBI32625.3	VIT_08s0040g03150.t01	Cytosolic ascorbate peroxidase	1.39
D7SKR5 (D7SKR5_VITVI)	CBI16243.3	VIT_06s0004g03550.t01	L-ascorbate peroxidase 2, cytosolic	1.39
D7UDY0 (D7UDY0_VITVI)	CBI40945.3	VIT_00s0260g00060.t01	FK506-binding protein 2-1	1.41
D7T674 (D7T674_VITVI)	CBI25995.3	VIT_05s0020g00600.t01	1-Cys peroxiredoxin	1.47
F6HIE6 (F6HIE6_VITVI)	CBI28862.3	VIT_12s0059g01320.t01	O-Glycosyl hydrolases family 17 protein	1.67
D7TC92 (D7TC92_VITVI)	CBI27750.3	VIT_11s0016g00420.t01	Protein Red isoform 1	2.01
**mRNA-splicing/Stability/Editing (n = 42)**				
D7ST85 (D7ST85_VITVI)	CBI19999.3	VIT_12s0055g00360.t01	Pinin/SDK/memA protein	1.39
F6HR01 (F6HR01_VITVI)	CBI32700.3	VIT_08s0040g02300.t01	DEAD-box ATP-dependent RNA helicase 57	1.45
D7TUX6 (D7TUX6_VITVI)	CBI34301.3	Not available	DCD (Development and Cell Death) domain protein	1.46
F6GUY6 (F6GUY6_VITVI)	CBI16084.3	VIT_06s0004g05220.t01	Serine/arginine repetitive matrix protein	1.50
D7SWX8 (D7SWX8_VITVI)	CBI21778.3	VIT_19s0027g00590.t01	RNA-binding protein with serine-rich domain-containing protein	1.52
A5AII6 (A5AII6_VITVI)	CBI37603.3	VIT_04s0044g00540.t01	Pre-mRNA-splicing factor ISY1 homolog isoform 1	1.63
E0CRK0 (E0CRK0_VITVI)	CBI19154.3	VIT_18s0001g05550.t01	Splicing factor, arginine/serine-rich	1.68
F6HC22 (F6HC22_VITVI)	CBI25319.3	VIT_13s0067g03600.t01	Arginine/serine-rich splicing factor	1.86
D7TBV2 (D7TBV2_VITVI)	CBI28137.3	VIT_11s0016g04610.t01	RNA-binding protein 25	1.74
D7TJ87 (D7TJ87_VITVI)	CBI30313.3	VIT_08s0007g03130.t01	Small nuclear ribonucleoprotein G	1.74
F6GSZ6 (F6GSZ6_VITVI)	CBI15706.3	VIT_17s0000g00960.t01	Omega-hydroxypalmitate O-feruloyl transferase	1.76
F6HZ42 (F6HZ42_VITVI)	CBI26627.3	VIT_07s0005g00320.t01	DEAD-box ATP-dependent RNA helicase 32	1.93
F6GU40 (F6GU40_VITVI)	CBI16368.3	VIT_06s0004g02220.t01	Heterogeneous nuclear ribonucleoprotein F-like	1.93
F6GUK3 (F6GUK3_VITVI)	CBI16510.3	VIT_06s0004g00710.t01	SC35-like splicing factor 33	1.21
A5AES3 (A5AES3_VITVI)	CBI24269.3	VIT_06s0009g02770.t01	Pre-mRNA branch site p14-like protein	1.61
F6GXF2 (F6GXF2_VITVI)	CBI17819.3	VIT_11s0052g01130.t01	CD2 antigen cytoplasmic tail-binding protein	1.31
F6HYI9 (F6HYI9_VITVI)	CBI36522.3	VIT_11s0037g00130.t01	FIP1[V]-like protein	1.81
D7T5U0 (D7T5U0_VITVI)	CBI25873.3	VIT_00s0625g00040.t01	Polyadenylate-binding protein 2	1.41
F6HTT9 (F6HTT9_VITVI)	CBI34206.3	VIT_14s0030g01680.t01	MKI67 FHA domain-interacting nucleolar phosphoprotein	1.27
D7TU07 (D7TU07_VITVI)	CBI33922.3	VIT_02s0012g00850.t01	Pre-mRNA-splicing factor CWC26	1.29
F6HYH6 (F6HYH6_VITVI)	CBI36502.3	VIT_04s0159g00020.t01	Polyadenylate-binding protein	1.28
D7TLV0 (D7TLV0_VITVI)	CBI31687.3	VIT_13s0019g01060.t01	Serine/arginine rich splicing factor	1.32
D7SJN7 (D7SJN7_VITVI)	CBI15863.3	VIT_06s0004g07530.t01	Spliceosomal protein	1.35
F6H257 (F6H257_VITVI)	CBI20322.3	VIT_19s0014g02920.t01	Pentatricopeptide repeat-containing protein	1.28
D7T3P2 (D7T3P2_VITVI)	CBI25124.3	VIT_03s0088g00390.t01	DnaJ homolog subfamily C member 17-like	1.25
F6GWL6 (F6GWL6_VITVI)	CBI17355.3	VIT_04s0023g03630.t01	Pre-mRNA-splicing factor CWC25	1.29
F6H2X4 (F6H2X4_VITVI)	CBI20826.3	VIT_04s0008g03130.t01	Pre-mRNA-splicing factor CWC21-like	1.30
D7TT33 (D7TT33_VITVI)	CBI33655.3	VIT_14s0006g02960.t01	Poly C-binding protein	0.36
F6I0Z0 (F6I0Z0_VITVI)	CBI37849.3	VIT_03s0038g02620.t01	Splicing factor	1.39
F6GYT6 (F6GYT6_VITVI)	CBI18525.3	VIT_18s0117g00150.t01	Heterogeneous nuclear ribonucleoprotein 27C	1.44
F6HP66 (F6HP66_VITVI)	CBI31839.3	VIT_16s0100g00450.t01	Arginine/serine-rich-splicing factor RSP40	1.44
D7UAL8 (D7UAL8_VITVI)	CBI39783.3	VIT_19s0015g00980.t01	Pre-mRNA-splicing factor cwc15	1.45
D7TAD5 (D7TAD5_VITVI)	CBI27458.3	VIT_01s0010g01410.t01	RNA-binding protein-like	1.53
F6I0P5 (F6I0P5_VITVI)	CBI37715.3	VIT_03s0038g04130.t01	DEAD-box ATP-dependent RNA helicase 42-like	1.56
F6H177 (F6H177_VITVI)	CBI19367.3	VIT_18s0001g08680.t01	Pre-mRNA-processing protein 40B	1.62
F6GTQ4 (F6GTQ4_VITVI)	CBI14910.3	VIT_17s0000g09680.t01	31 kDa ribonucleoprotein	1.67
F6GWX4 (F6GWX4_VITVI)	CBI17535.3	VIT_04s0023g01580.t01	U1 small nuclear ribonucleoprotein 70 kDa	1.70
F6HI04 (F6HI04_VITVI)	CBI28632.3	VIT_04s0043g00270.t01	Pre-mRNA-splicing factor 38B	1.72
F6I0K0 (F6I0K0_VITVI)	CBI37648.3	VIT_04s0044g00080.t01	Heterogeneous nuclear ribonucleoprotein F	1.78
D7UD56 (D7UD56_VITVI)	CBI40671.3	VIT_11s0078g00440.t01	U4/U6.U5 tri-snRNP-associated protein	1.82
F6HF25 (F6HF25_VITVI)	CBI27081.3	VIT_01s0011g02820.t01	Protein decapping 5 isoform X1	1.85
F6HTK3 (F6HTK3_VITVI)	CBI34075.3	VIT_03s0017g01340.t01	Heterogeneous nuclear ribonucleoprotein Q	2.10
**rRNA processing/Biogenesis (n = 13)**				
D7T103 (D7T103_VITVI)	CBI24130.3	VIT_19s0085g01090.t01	Nuclear-pore anchor-like	1.29
E0CQ61 (E0CQ61_VITVI)	CBI19866.3	VIT_18s0001g14320.t01	Nucleolar protein 58 isoform X1	1.52
F6GST5 (F6GST5_VITVI)	CBI15641.3	VIT_17s0000g01640.t01	RNA-metabolising metallo-beta-lactamase family protein	1.93
F6H683 (F6H683_VITVI)	CBI22501.3	VIT_03s0091g00320.t01	Ribosomal RNA assembly protein mis3-like	0.72
F6HLD3 (F6HLD3_VITVI)	CBI30568.3	VIT_08s0007g00190.t01	H/ACA ribonucleoprotein complex subunit 4	1.45
D7T1S3 (D7T1S3_VITVI)	CBI24453.3	VIT_00s0264g00120.t01	Scaffold attachment factor B1	1.91
C5DB53 (C5DB53_VITVI)	CBI31135.3	VIT_08s0056g00160.t01	U3 small nucleolar RNA-associated protein 11	1.34
F6GZQ7 (F6GZQ7_VITVI)	CBI16359.3	VIT_18s0001g13560.t01	Midasin	1.21
F6HF03 (F6HF03_VITVI)	CBI27323.3	VIT_01s0011g00070.t01	Nucleolar protein 14-like	1.50
D7STQ8 (D7STQ8_VITVI)	CBI20657.3	VIT_04s0008g01200.t01	Translation machinery-associated protein 22 isoform 2	0.33
F6I6B2 (F6I6B2_VITVI)	CBI40495.3	VIT_15s0046g01120.t01	Ribosome biogenesis regulatory protein homolog	1.58
D7U276 (D7U276_VITVI)	CBI36842.3	VIT_07s0005g00270.t01	Nucleolar protein 16 involved in ribosome biogenesis	1.90
F6GXL7 (F6GXL7_VITVI)	CBI17936.3	VIT_07s0141g00380.t01	U3 small nucleolar RNA-associated protein-like protein	2.00
**Stress responses (n = 13)**				
F6HS56 (F6HS56_VITVI)	CBI33350.3	VIT_05s0051g00650.t01	Voltage-gated potassium channel subunit beta	0.72
E0CUG6 (E0CUG6_VITVI)	CBI22747.3	VIT_16s0050g00140.t01	Metal ion binding protein	1.63
F6GY60 (F6GY60_VITVI)	CBI18236.3	VIT_18s0072g00160.t01	Peroxidase 12-like	1.34
D7TUZ6 (D7TUZ6_VITVI)	CBI34321.3	VIT_14s0030g00430.t01	Prefoldin chaperone subunit family protein	1.21
E0CRL1 (E0CRL1_VITVI)	CBI19165.3	VIT_18s0001g05720.t01	14-3-3 protein 7	1.27
F6H0X3 (F6H0X3_VITVI)	CBI19195.3	VIT_18s0001g06330.t01	14-3-3 protein	1.49
F6H824 (F6H824_VITVI)	CBI23432.3	VIT_00s0250g00040.t01	DNA-binding protein	1.21
D7SU28 (D7SU28_VITVI)	CBI20777.3	VIT_04s0008g02590.t01	Selenium binding protein	1.22
F6HEA6 (F6HEA6_VITVI)	CBI26439.3	VIT_16s0039g01020.t01	Adenylate cyclase, terminal-differentiation specific	1.30
D7TIR0 (D7TIR0_VITVI)	CBI30136.3	Not available	Arginine/serine-rich coiled-coil protein 2 isoform X2	1.43
F6H1I0 (F6H1I0_VITVI)	CBI19683.3	VIT_18s0001g12350.t01	Protein ESSENTIAL FOR POTEXVIRUS ACCUMULATION X1	1.59
F6H2Z1 (F6H2Z1_VITVI)	CBI20901.3	Not available	Dehydration-responsive protein RD22	1.93
F6HE42 (F6HE42_VITVI)	CBI26016.3	VIT_05s0020g00840.t01	Late embryogenesis abundant protein D-29	4.17
**Transcriptional regulation (n = 20)**				
F6H7R2 (F6H7R2_VITVI)	CBI23284.3	VIT_07s0197g00070.t01	Upstream activation factor subunit spp27-like	1.59
D7SIK8 (D7SIK8_VITVI)	CBI15319.3	VIT_17s0000g05190.t01	Zinc finger CCCH domain-containing protein	1.64
D7TCU3 (D7TCU3_VITVI)	CBI28316.3	VIT_06s0080g00460.t01	Nuclear transcription factor Y subunit B-8	1.71
D7TDY1 (D7TDY1_VITVI)	CBI28704.3	VIT_07s0151g00910.t01	NF-kappa-B-activating protein	1.21
F6HSW0 (F6HSW0_VITVI)	CBI33736.3	VIT_07s0129g00610.t01	FRIGIDA-like isoform 2	1.28
F6I111 (F6I111_VITVI)	CBI37898.3	VIT_03s0038g02130.t01	Cold-shock DNA binding protein	1.47
D7UDF0 (D7UDF0_VITVI)	CBI40765.3	Not available	AT-hook protein 1	1.36
D7SK51 (D7SK51_VITVI)	CBI16027.3	VIT_06s0004g05830.t01	DNA-directed RNA polymerases I and III subunit RPAC2 isoform 1	1.25
F6HZB5 (F6HZB5_VITVI)	CBI36973.3	VIT_07s0005g01740.t01	Zinc knuckle (CCHC-type) family protein	1.37
F6HIW1 (F6HIW1_VITVI)	CBI29150.3	VIT_13s0047g00310.t01	Serrate RNA effector molecule-like	1.37
E0CNQ9 (E0CNQ9_VITVI)	CBI19287.3	VIT_18s0001g07750.t01	Neuroguidin-like	1.38
F6HAX1 (F6HAX1_VITVI)	CBI24668.3	VIT_05s0094g00440.t01	Sas10/U3 ribonucleoprotein family protein	1.42
D7SII5 (D7SII5_VITVI)	CBI15296.3	VIT_17s0000g05450.t01	Early flowering 5 protein	1.43
D7TZU6 (D7TZU6_VITVI)	CBI35892.3	VIT_09s0002g01530.t01	GBF-interacting protein 1-like isoform X1	1.52
F6HH48 (F6HH48_VITVI)	CBI28116.3	VIT_11s0016g04390.t01	Nucleolar protein dao-5-like	1.54
F6HFZ8 (F6HFZ8_VITVI)	CBI27460.3	VIT_01s0010g01440.t01	Transcription elongation regulator 1-like	1.57
D7TTQ2 (D7TTQ2_VITVI)	CBI33817.3	VIT_02s0012g02250.t01	Transcription factor HBP-1a	1.69
F6I758 (F6I758_VITVI)	CBI40894.3	VIT_13s0175g00120.t01	ABSCISIC ACID-INSENSITIVE 5-like protein 2-like isoform 1	1.75
F6HLJ6 (F6HLJ6_VITVI)	CBI30026.3	VIT_08s0007g06400.t01	Zinc finger CCCH domain-containing protein 14-like isoform 1	1.83
D7SIC5 (D7SIC5_VITVI)	CBI15235.3	VIT_17s0000g06060.t01	Activating signal cointegrator 1	2.00

## Data Availability

The mass spectrometry proteomics data have been deposited to the ProteomeXchange Consortium via the PRIDE [1] partner repository with the dataset identifier PXD004988 and nul.

## Supplementary Materials

The following are available online at https://www.mdpi.com/article/10.3390/ijms23031537/s1.
