# Supplementary material for: Grape ASR-Silencing Sways Nuclear Proteome, Histone Marks and Interplay of Intrinsically Disordered Proteins
Source: Int J Mol Sci. 2022 Jan 28;23(3):1537. doi: 10.3390/ijms23031537 (PMC8835812; doi:10.3390/ijms23031537)
Supplement: Supplementary file 1 [file ijms-23-01537-s001.zip › ijms-1543182-supplementary.pdf]

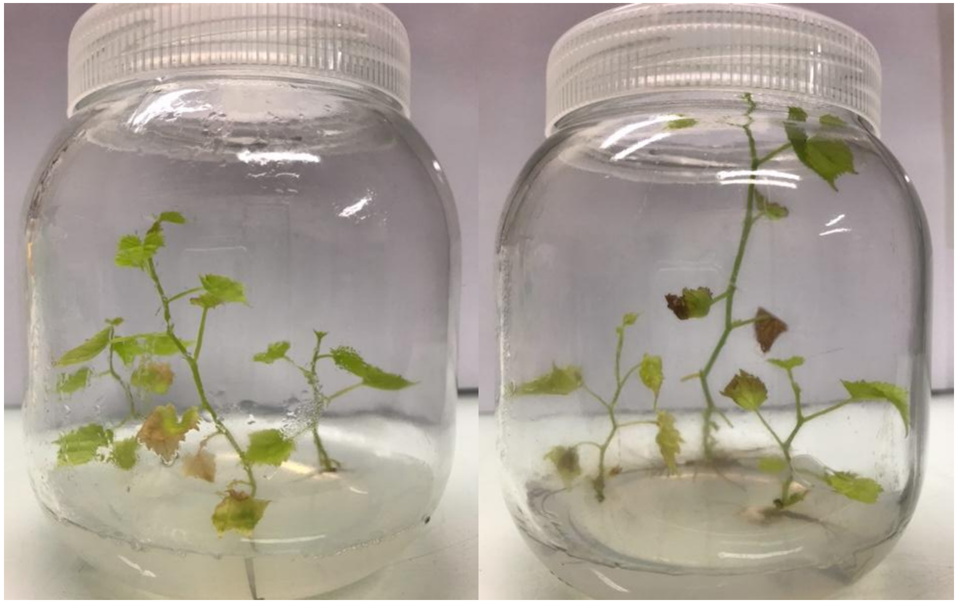

In vitro regenerated wild-type (Left) and transgenic VvMSA-RNAi silenced (Right) planlets

Figure S1

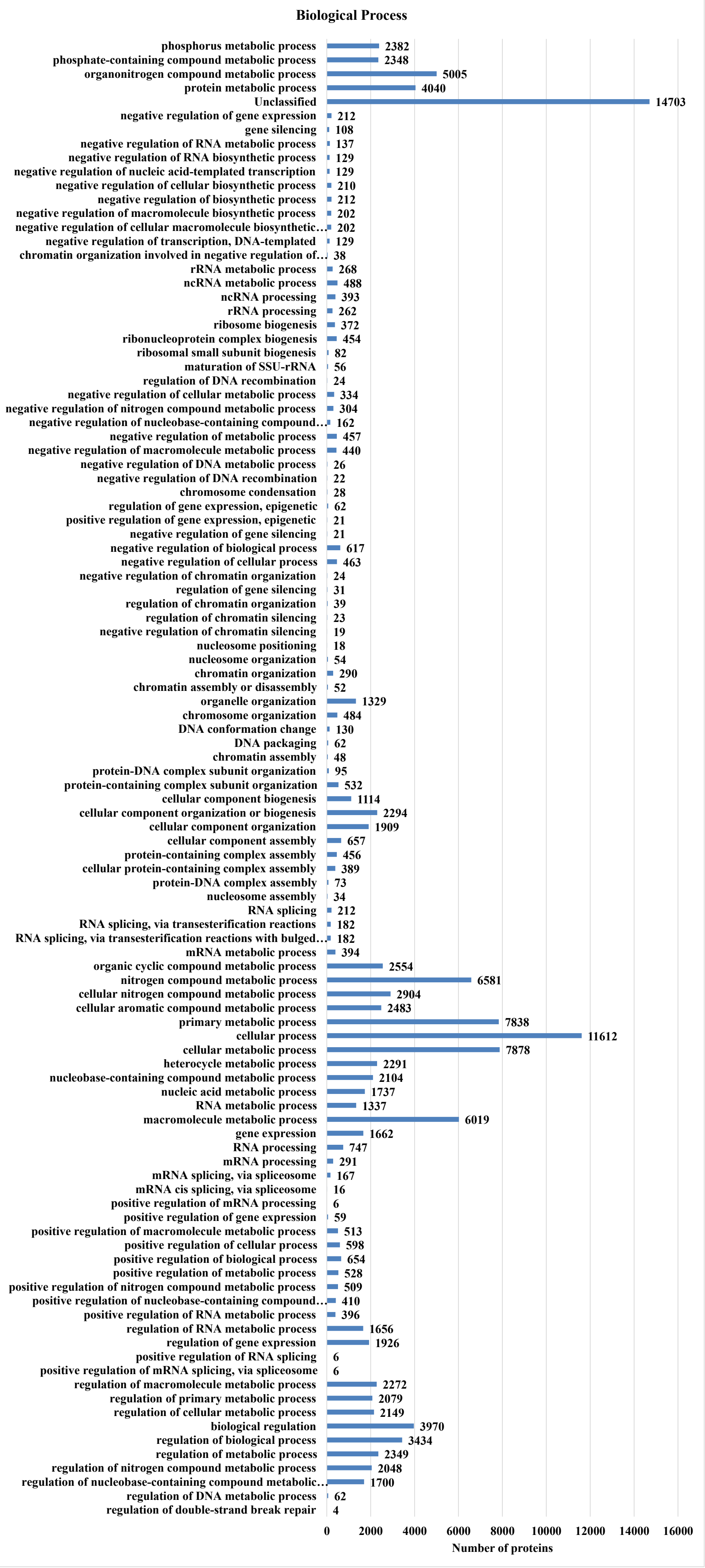
Biological Process
phosphorus metabolic process 2382
phosphate-containing compound metabolic process 2348
organonitrogen compound metabolic process 5005
protein metabolic process 4040
Unclassified 14703
negative regulation of gene expression 212
gene silencing 108
negative regulation of RNA metabolic process 137
negative regulation of RNA biosynthetic process 129
negative regulation of nucleic acid-templated transcription 129
negative regulation of cellular biosynthetic process 210
negative regulation of biosynthetic process 212
negative regulation of macromolecule biosynthetic process 202
negative regulation of cellular macromolecule biosynthetic… 202
negative regulation of transcription, DNA-templated 129
chromatin organization involved in negative regulation of… 38
rRNA metabolic process 268
ncRNA metabolic process 488
ncRNA processing 393
rRNA processing 262
ribosome biogenesis 372
ribonucleoprotein complex biogenesis 454
ribosomal small subunit biogenesis 82
maturation of SSU-rRNA 56
regulation of DNA recombination 24
negative regulation of cellular metabolic process 334
negative regulation of nitrogen compound metabolic process 304
negative regulation of nucleobase-containing compound… 162
negative regulation of metabolic process 457
negative regulation of macromolecule metabolic process 440
negative regulation of DNA metabolic process 26
negative regulation of DNA recombination 22
chromosome condensation 28
regulation of gene expression, epigenetic 62
positive regulation of gene expression, epigenetic 21
negative regulation of gene silencing 21
negative regulation of biological process 617
negative regulation of cellular process 463
negative regulation of chromatin organization 24
regulation of gene silencing 31
regulation of chromatin organization 39
regulation of chromatin silencing 23
negative regulation of chromatin silencing 19
nucleosome positioning 18
nucleosome organization 54
chromatin organization 290
chromatin assembly or disassembly 52
organelle organization 1329
chromosome organization 484
DNA conformation change 130
DNA packaging 62
chromatin assembly 48
protein-DNA complex subunit organization 95
protein-containing complex subunit organization 532
cellular component biogenesis 1114
cellular component organization or biogenesis 2294
cellular component organization 1909
cellular component assembly 657
protein-containing complex assembly 456
cellular protein-containing complex assembly 389
protein-DNA complex assembly 73
nucleosome assembly 34
RNA splicing 212
RNA splicing, via transesterification reactions 182
RNA splicing, via transesterification reactions with bulged… 182
mRNA metabolic process 394
organic cyclic compound metabolic process 2554
nitrogen compound metabolic process 6581
cellular nitrogen compound metabolic process 2904
cellular aromatic compound metabolic process 2483
primary metabolic process 7838
cellular process 11612
cellular metabolic process 7878
heterocycle metabolic process 2291
nucleobase-containing compound metabolic process 2104
nucleic acid metabolic process 1737
RNA metabolic process 1337
macromolecule metabolic process 6019
gene expression 1662
RNA processing 747
mRNA processing 291
mRNA splicing, via spliceosome 167
mRNA cis splicing, via spliceosome 16
positive regulation of mRNA processing 6
positive regulation of gene expression 59
positive regulation of macromolecule metabolic process 513
positive regulation of cellular process 598
positive regulation of biological process 654
positive regulation of metabolic process 528
positive regulation of nitrogen compound metabolic process 509
positive regulation of nucleobase-containing compound… 410
positive regulation of RNA metabolic process 396
regulation of RNA metabolic process 1656
regulation of gene expression 1926
positive regulation of RNA splicing 6
positive regulation of mRNA splicing, via spliceosome 6
regulation of macromolecule metabolic process 2272
regulation of primary metabolic process 2079
regulation of cellular metabolic process 2149
biological regulation 3970
regulation of biological process 3434
regulation of metabolic process 2349
regulation of nitrogen compound metabolic process 2048
regulation of nucleobase-containing compound metabolic… 1700
regulation of DNA metabolic process 62
regulation of double-strand break repair 4
0 2000 4000 6000 8000 10000 12000 14000 16000
Number of proteins

Isolation and purification of nuclei from grape 41B embryogenic cells

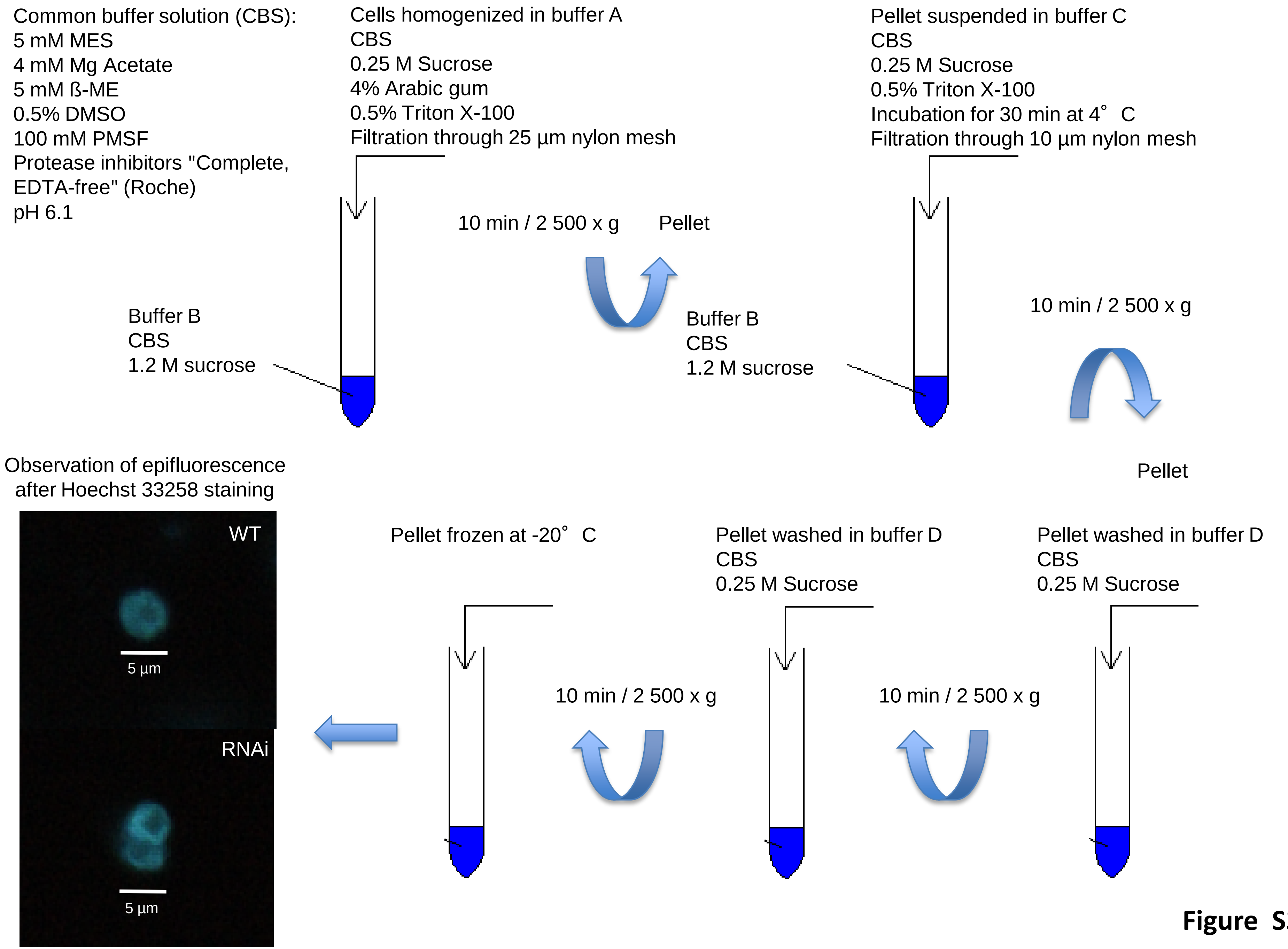

Figure S3

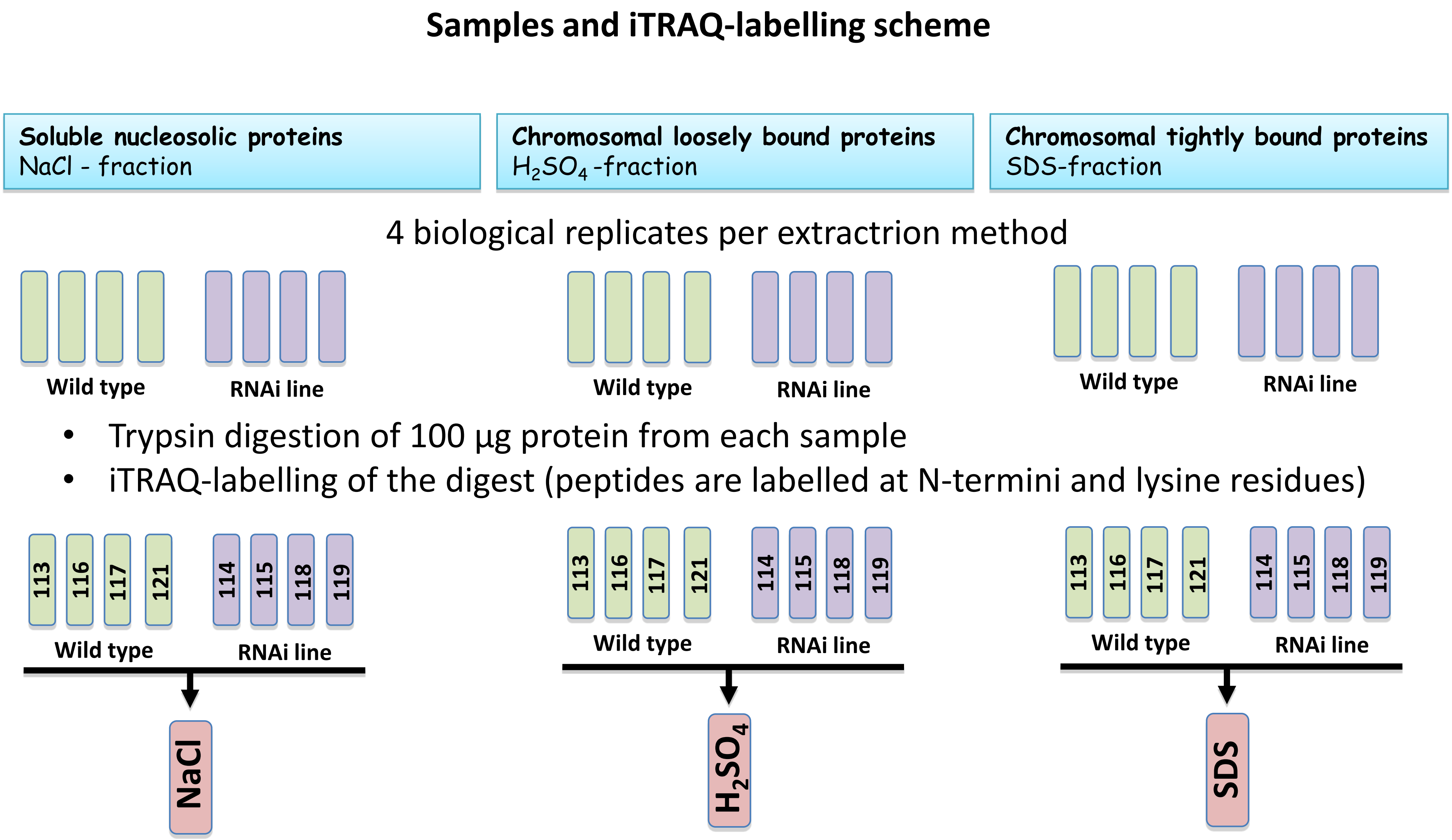
Samples and iTRAQ-labelling scheme
Soluble nucleosolic proteins
NaCl - fraction
Chromosomal loosely bound proteins
H2SO4 -fraction
Chromosomal tightly bound proteins
SDS-fraction
4 biological replicates per extractrion method
Wild type
RNAi line
Wild type
RNAi line
Wild type
RNAi line
• Trypsin digestion of 100 µg protein from each sample
• iTRAQ-labelling of the digest (peptides are labelled at N-termini and lysine residues)
113
116
117
121
114
115
118
119
Wild type
RNAi line
NaCl
113
116
117
121
114
115
118
119
Wild type
RNAi line
H2SO4
113
116
117
121
114
115
118
119
Wild type
RNAi line
SDS

Figure S4

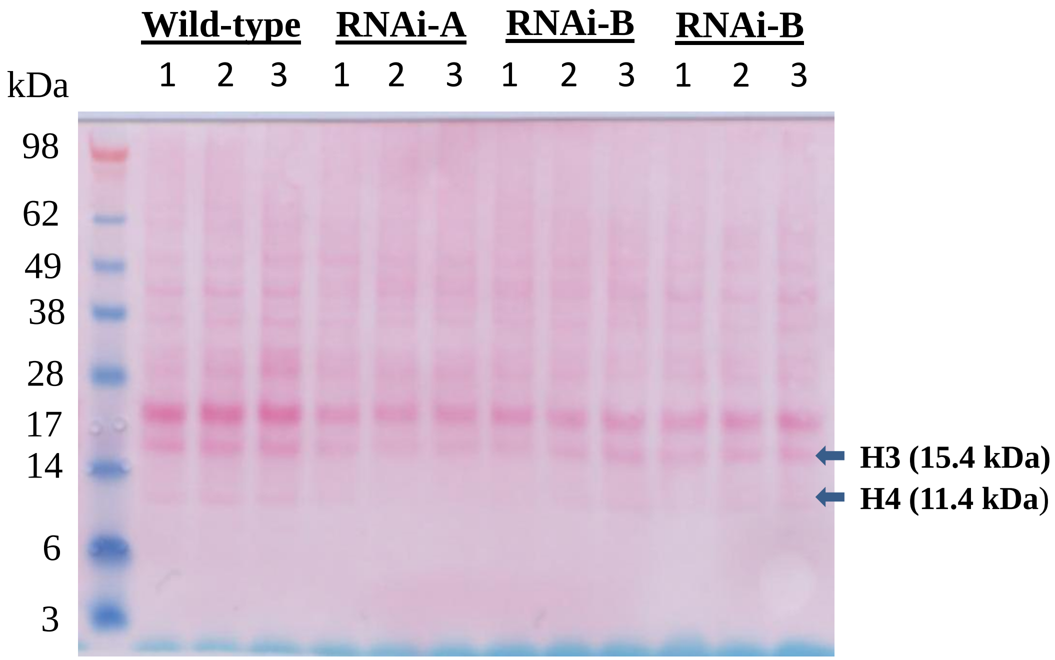

Supplement to Fig. 4A - Loading controls (Ponceau S staining)

On the left : SeeBlue Plus2 Pre-stained Protein Standard, LC 5925 (Thermo Fisher Scientific)
Calculated mass (Da) of predicted histone sequences (*Vitis vinifera*):
- Histone H3 (*V. vinifera*): 15 268 Da (136 AA), 15 406 Da (136 AA), 15 537 AA (137 AA) ; Mean = 15403,66 ≈ 15.4 kDa
- Histone H4 (*V. vinifera*): 11 409 Da (103 AA) ≈ 11.4 kDa

Figure S5

Table S1 - List of antibodies

Histone mAbs (mouse)		Provider, Cat. ≠	Stock Conc. (µg/µL)	Immunoblot Dilution
1	**Histone H3**	Abcam, 24834	0.50	0.25 µg/mL
2	**Histone H4**	Abcam, 17036	0.50	0.50 µg/mL
**Histone H3 PTM mAbs (mouse)**		**Provider, Cat. ≠**	**Stock Conc. (µg/µL)**	**Immunoblot Dilution**
1	**H3K4me1**	Active Motif, 39635	0.64	1: 4 000
2	**H3K4me2**	Active Motif, 39679	1.00	2 µg/mL
3	**H3K4me3**	Active Motif, 61379	0.72	1 µg/mL
4	**H3K9ac**	Active Motif, 61251	1.00	2 µg/mL
5	**H3K9me1**	Active Motif, 39681	0.40	1 µg/mL
6	**H3K9me2**	Active Motif, 39683	0.72	2 µg/mL
7	**H3K14ac**	Active Motif, 61433	0.73	2 µg/mL
8	**H3K27ac**	Active Motif, 39685	1.00	2 µg/mL
9	**H3K27me1**	Active Motif, 61015	0.56	0.50 µg/mL
10	**H3K27me3**	Active Motif, 61017	0.77	0.50 µg/mL
11	**H3K36me2**	Active Motif, 61019	0.75	2 µg/mL
12	**H3K36me3**	Active Motif, 61021	0.75	1 µg/mL
**Histone H4 PTM mAbs (mouse)**		**Provider, Cat. ≠**	**Stock Conc. (µg/µL)**	**Immunoblot Dilution**
1	**H4K5ac**	Active Motif, 61523	1.00	2 µg/mL
2	**H4K8ac**	Active Motif, 61525	0.62	2 µg/mL
3	**H4K12ac**	Active Motif, 61527	0.72	2 µg/mL
4	**H4K16ac**	Active Motif, 61529	0.70	2 µg/mL
5	**H4K20ac**	Active Motif, 61531	0.52	2 µg/mL
6	**H4K20me1**	Active Motif, 39727	1.00	1 µg/mL

Table S2. Immunoblotting materials and user manuals

NuPAGE® LDS Sample Buffer (4X), 10 mL, ref. NP0007
NuPAGE® MES SDS Running Buffer (20X), 500 mL, ref. NP0002
NuPAGE™ Midi Gel System (User Guide, Pub. No. MAN0006268, 2017)
NuPAGE® Sample Reducing Agent (10X), 10 mL, ref. NP0009
Power Blotter Cassette XL, ref. PB003
Power Blotter Select Transfer Stacks (Pub. No. MAN0017051, 2017)
Power Blotter System (User Guide, Pub. No. MAN0017053, 2017)
SeeBlue® Plus2 Prestained Protein Standard, 500 µL, ref. LC5925
XCell4 SureLock™ Midi-Cell (User Guide, Pub. No. MAN0000534, 2014)

Table S3. RNAi/WT ratios used for generation of the heat map on Figure 4C

Ratio	H3K4me1	H3K4me2	H3K4me3	H3K9ac	H3K9me1	H3K9me2	H3K14ac	H3K27me1	H3K27me3	H3K36me2	H3K36me3	H4K16ac*	H4K20ac
**RNAi-A/WT**	0,59	1,20	0,75	0,51	0,76	1,49	0,84	0,81	1,17	0,27	0,72	0,00	1,40
**RNAi-B/WT**	0,74	1,56	0,69	0,93	0,33	1,79	0,72	1,01	1,18	0,28	0,77	0,00	1,08
**RNAi-C/WT**	0,68	1,12	0,60	1,06	0,45	2,38	1,47	0,68	0,65	0,22	0,55	0,00	1,35
Mean	***0,67***	***1,29***	***0,68***	***0,84***	***0,51***	***1,89***	***1,01***	***0,83***	***1,00***	***0,26***	***0,68***	***0,00***	***1,28***
